# Design Compact Absorptive Common-Mode Noise Suppression Filter with Series Unified Circuit

**DOI:** 10.3390/s23020957

**Published:** 2023-01-14

**Authors:** Cheng-Yi Zhuang, Ding-Bing Lin

**Affiliations:** Department of Electronic and Computer Engineering, National Taiwan University of Science and Technology, Taipei City 106, Taiwan

**Keywords:** common-mode noise filter (CMNF), common-mode noise absorption, radio frequency interference (RFI), signal integrity (SI), electromagnetic interference (EMI)

## Abstract

In the PCB process, overcoming common-mode noise radiation is critical. In past years, most studies have focused on a common-mode noise filter (CMNF) that can solve electromagnetic interference in high-speed digital systems by blocking and absorbing common-mode noise radiation. Unfortunately, connecting with any reflective common-mode noise filter (R-CMNF) and reducing the area of an absorptive common-mode noise filter (A-CMNF) are the most troublesome tasks in the PCB process. A novel equivalent circuit is proposed in this research to minimize the complexity of the design and improve accuracy. Detailed analyses of this proposed approach are entirely depicted in this article. The experiment result shows that 9% of fractional bandwidth centered at 2.25 Hz can achieve at least 90% absorption efficiency. With our proposed method, the area of A-CMNF is smaller than in state-of-the-art research.

## 1. Introduction

Now is the era of high-speed digital signal transmission, and the dominant transmission uses differential signal transmission lines. Suppose single-ended signals are still used to transmit at high frequencies. In that case, the signal will produce serious crosstalk, so the differential signal is used as the signal transmission method in most high-speed digital circuit systems.

However, when the differential signal is transmitted on a printed circuit board (PCB), there are still many problems caused by unsatisfactory characteristics, such as inhomogeneous dielectric, unequal time delays, or the rise and fall time of complementary signals that are inconsistent. The above problems are unavoidable and cause mode conversion—the signal changes from differential mode to common mode. In addition, the common-mode signal is also called “common mode noise” because the return path of the common-mode signal contains the discontinuous part of the impedance of the connector end, or the outer conductor of the cable, which will generate radiation and affect the nearby antenna or radio frequency components [1,2].

Various methods have been proposed in the existing literature to solve radiation caused by common-mode signals, such as adding absorbing materials to the coupling path to suppress noise radiation [3,4,5]. However, this method is more suitable for large electronic products because the absorbing material needs enough space to be placed. Another way is using the strip line to design a circuit in which the dielectric medium is more uniform than the microstrip line and covered by the reference plane so that the performance is better than microstrip lines [6]. However, using this method will increase the product cost and the number of layers of the circuit board. Thus, that is only used in specific areas, and most are still microstrip line traces. Using a common-mode choke coil is another common way because it easily applies to different applications. However, when it comes to 5G mobile communications or Wi-Fi 6, more and more challenges are encountered. The increase in frequency bands and more comprehensive bandwidth makes frequency bands overlap between digital and wireless signals. However, the common-mode choke coil only suppresses a single band.

A common-mode noise filter (CMNF) is now proposed to solve the problem of RFI and have a better effect than the technologies mentioned above. This method can solve the problem of the embedded absorbing material occupying too much space and can be made into components and placed in the circuit. Otherwise, the circuit design can suppress the common-mode noise of different frequency bands, which solves the disadvantage of common-mode chokes that only work at a single band. Many common-mode noise filter architectures have been proposed in recent research [7,8,9,10,11,12,13,14,15,16,17,18], but although these designs can effectively suppress common-mode noise, they still occupy a large area.

Therefore, a novel design method is proposed in this paper. The technique can significantly reduce the area of CMNF, and the overall structure is simple. Otherwise, it is suitable for making small components and can be flexibly used in different circuits. The following content includes circuit analysis and validation. Section 2 introduces the design concepts of common-mode noise filters and shows how to use the matching series Unified Circuit to design absorptive common-mode noise filters. Section 3 will verify the proposed design using FEM simulation software, presenting the simulation and measurement results. Finally, we summarize the proposed architecture features and compare the area with the referenced works, which are the single band and unidirectional A-CMNF.

## 2. Methodology

### 2.1. Common-Mode Noise Filter Design Concepts

The high-speed signal on differential pairs is composed of the common- and differential-mode signals. The common-mode signal of zero is ideal, but mode conversion is unavoidable because of layout requirements or timing skews. Then, the common-mode signal converted from the differential-mode signal will radiate at the impedance mismatching part, such as the connector. Hence, the common-mode noise filter blocks the common-mode signal by adding a band-pass filter structure at the return path and still keeps the differential-mode signal integrity.

Under odd-mode analysis, the differential-mode signal is out of phase, and there is an electric wall in the middle of the circuit. It can be considered an ideal return path for the differential-mode signal. Under even-mode analysis, the common-mode signal is blocked by the band-pass filter, which usually is a parallel *LC*-tank resonator. The parallel *LC*-tank resonator is placed at the return path and works as an open circuit at the target band to break the common-mode signal path. This way is the so-called reflective common-mode noise filter (R-CMNF) [7,16,17].

Impedance matching is the other most important issue, whether in differential or common mode. When the differential signal is transmitted, the odd mode’s characteristic impedance must be maintained to preserve signal integrity if the signal passes through a different reference plane. Otherwise, no reflection is expected in the common mode because high reflection will cause unexpected electromagnetic interference. However, as previously stated, R-CMNF is a high-reflection circuit because it blocks common-mode signal transmission. Hence, an absorptive common-mode noise filter (A-CMNF) is later proposed, which has block and absorption features in common mode [8,9,10,11,12,13,14,15,18]. The following section will introduce a novel design method of A-CMNF and analyze the equivalent circuit.

### 2.2. Proposed Methodology

**Figure 1:**
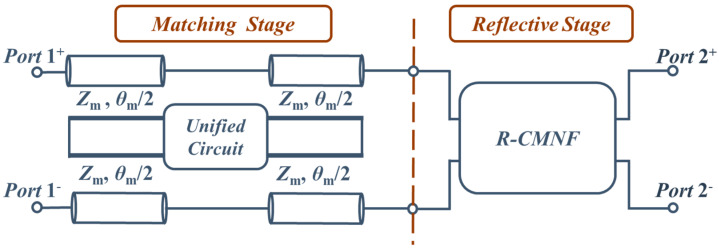
Schematic of the proposed structure.

Figure 1 is the proposed structure composed of two stages, the Reflective Stage and the Absorptive Stage, and it is a four-terminals circuit. First, The Reflective Stage is used to block the common-mode noise. The Matching Stage, which consists of two transmission lines with a lumped Unified Circuit, makes the input impedance of the reflective stage match the even-mode characteristic impedance of the feeding differential pair.

**Figure 2:**
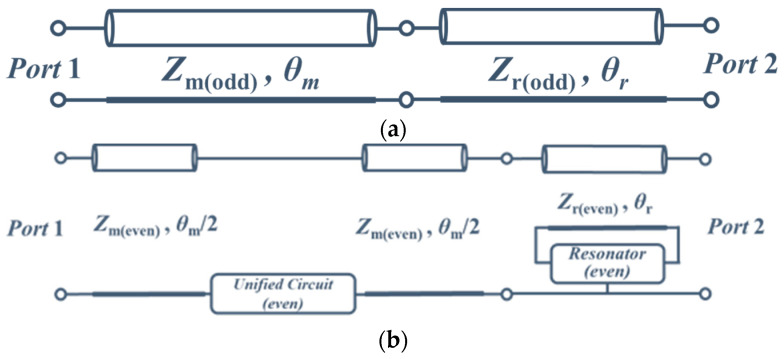
(**a**) Odd-mode equivalent half circuit of proposed structures; (**b**) Even-mode equivalent half circuit of proposed structures.

Consider the odd-mode half circuit shown in Figure 2a. There is a uniform transmission with odd-mode characteristic impedance, *Z*_m(odd)_ and *Z*_r(odd)_, which usually are 50 Ω. The electrical length of the transmission line is *θ*_m_ + *θ*_r_.

Figure 2b presents the even-mode half circuit that includes a resonator and Unified Circuit. The Matching Stage is divided into two parts one is the connection part, and the other is the unified circuit part. The even-mode characteristic impedance is the same for both parts, and the electrical length is *θ*_m_. The input impedance of the Unified Circuit is *Z*_U_, which is undecided and ready for circuit design.

### 2.3. Equivalent Circuit of the Reflective Stage

**Figure 3:**
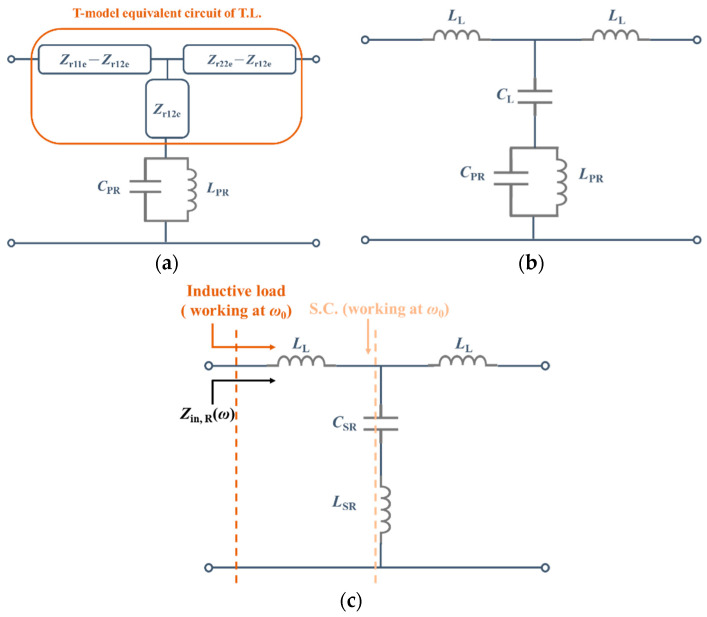
(**a**) The equivalent circuit of the Reflective Stage; (**b**) Use lumped component to represent the equivalent circuit; (**c**) The simplified equivalent circuit of the Reflective Stage.

Under even mode, the Reflective Stage can be transformed into a T-model equivalent circuit that cascades a parallel resonator. The impedance parameters of the T-model are shown in (1). The value of *C*_PR_ and *L*_PR_ depends on the structure, and the Matching Stage’s electrical length depends on the Unified Circuit’s interconnect structure and connection line. Additionally, if there is an even-mode characteristic impedance mismatch, it will be ignored here because there is no need to maintain common-mode signal integrity. Then the equivalent circuit model of the Reflective Stage is established, as shown in Figure 3a.

*Z*_r11e_ = *Z*_r22e_ = −*jZ*_r(even)_ cot(*θ*_r_), (1)

*Z*_r12e_ = *Z*_r21e_ = −*jZ*_r(even)_ csc(*θ*_r_), (2)

*Z*_r11e_ − *Z*_r12e_ = *jZ*_r(even)_ sin(*θ*_r_/2), (3)

Furthermore, the T-model circuit of a transmission line can be transformed into a lumped circuit when the electrical length is less than a quarter wavelength [19]. The lumped equivalent circuit is shown in Figure 3b. The *Z*_r12e_ becomes a capacitor, *C*_L_, and *Z*_r11e_*−Z*_r12e_ becomes an inductor, *L*_L_, where the values can be calculated by (4) and (5).

(4) $$L_{L}=\frac{Z_{r(\mathrm{even})}\sin(\theta_{r}/2)}{\omega },$$

(5) $$C_{L}=\frac{1}{\omega Z_{r(\mathrm{even})}\csc(\theta_{r})},$$

The *LC*-tank with the capacitor *C*_L_ can be converted to the *LC*-branch by the Kirchhoff Circuit Laws. Finally, the simplified equivalent circuit of the Reflective Stage is shown in Figure 3c, and the input impedance of the Reflective Stage can be written as (6). When working at resonance frequency *ω*_0_, the input impedance is only *jω*_0_*L*_L_ because the *LC*-branch is a short circuit at that time.

(6) $$Z_{\mathrm{in},\;R(\omega )}=j\omega L_{L}\;+[(\frac{1}{{j\omega C}_{\mathrm{SR}}}+j\omega L_{\mathrm{SR}})//j\omega L_{L}]$$

It is deserved to be noted that even if there are different Reflective Stage designs, such as mushroom-like structures [7], quarter-wavelength resonators [16], and E-type resonators [17], the proposed equivalent circuit model is still functional because the design mentioned above is considered to be the same as a differential pair with a parallel resonance circuit. This article uses a mushroom-like structure to validate the proposed method and tries to make compact sizes in the PCB process.

### 2.4. Design Procedures of The Matching Stage

**Figure 4:**
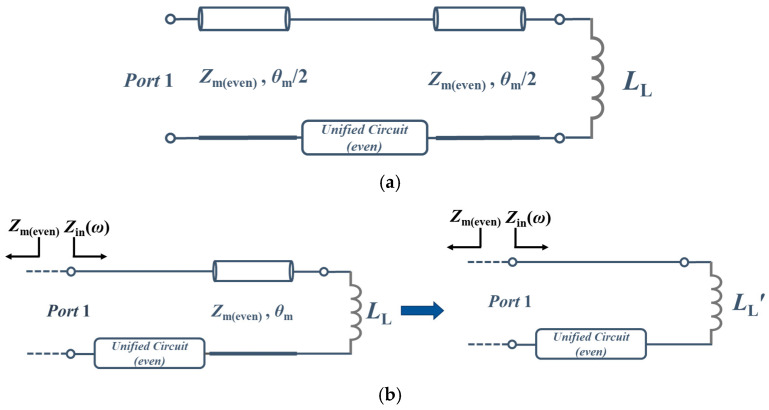
(**a**) The equivalent circuit of the whole structure; (**b**) Simplified Matching Stage with inductive load and use the inductor *L*_L_ʹ to alternate the inductor load *L*_L_ with a transmission line.

The Matching Stage is for performing impedance matching, letting the reflection of the whole structure be zero. According to equivalent circuit analysis, the R-CMNF is considered an inductor, LL, and the entire system can be represented in Figure 4a. The electrical length can be considered as *θ*_m_ to reduce the complexity of the analysis, and the Unified Circuit and the connection line that makes the electrical length of the Matching Stage can be clearly analyzed. Then, the structure will become straightforward to design the Unified Circuit, as shown in Figure 4b. The inductor *L*_L_ through a transmission line will change its inductance by using *L*_L_′ to describe the new inductance. The value of *L*_L_′ can be calculated using the Smith Chart or the input impedance equation.

To eliminate the reflection, the input impedance *Z*_in_(*ω*_0_) must be equal to *Z*_m(even)_, so there is a clear relation as (7), and *Z*_U_ describes the input impedance of the Unified Circuit. Please note that the following equation only works at the same frequency because using *L*_L_′ to replace the Reflective Stage only happens at the resonance frequency. Then, the Unified Circuit value can be calculated with (8).

**Figure 5:**
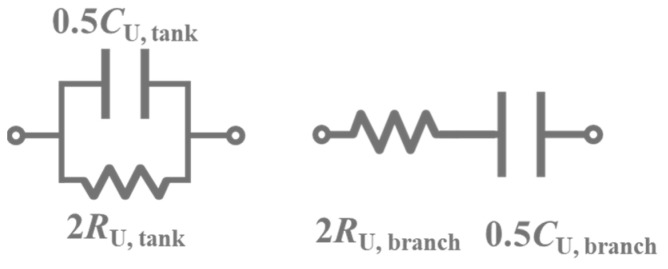
Two types of Unified Circuits. Left is the *RC*-tank, and right is the *RC*-branch.

By observation (8), the Unified Circuit must have a resistor and a capacitor that meet the impedance matching concept. There are two kinds of Unified Circuits, the *RC*-tank and *RC*-branch, as shown in Figure 5. The component values are represented under even mode, which is why the resistor is 2*R*_U_, and the capacitor is 0.5*C*_U_. In this article, we chose *RC*-tank as the Unified Circuit and validated it in the PCB process.

*Z*_m(even)_ = *Z*_U_(*ω*_0_) + *jω*_0_*L*_L_′ (7)

*Z*_U_(*ω*_0_) = *Z*_m(even)_ − *jω*_0_*L*_L_′ (8)

The specific component value can be calculated by (8), but if choosing *RC*-branch, there are different resistor and capacitor values to the *RC*-tank. Therefore, there are two equations to calculate tank and branch, represented in (9)–(12), respectively.

(9) $$R_{U,\;\mathrm{tank}}=\frac{{Z_{m(\mathrm{even})}}^{2}+{\left(\omega_{0}L_{L}\right)}^{2}}{2Z_{m(\mathrm{even})}}$$

(10) $$C_{U,\;\mathrm{tank}}=\frac{2L_{L}}{{Z_{m(\mathrm{even})}}^{2}+{\left(\omega_{0}L_{L}\right)}^{2}}$$

(11) $$R_{U,\;\mathrm{branch}}=\frac{Z_{m(\mathrm{even})\;}}{2}$$

(12) $$C_{U,\;\mathrm{branch}}=\frac{2L_{L}}{{Z_{m(\mathrm{even})}}^{2}+{\left(\omega_{0}L_{L}\right)}^{2}}$$

## 3. Validation and Measurement

**Figure 6:**
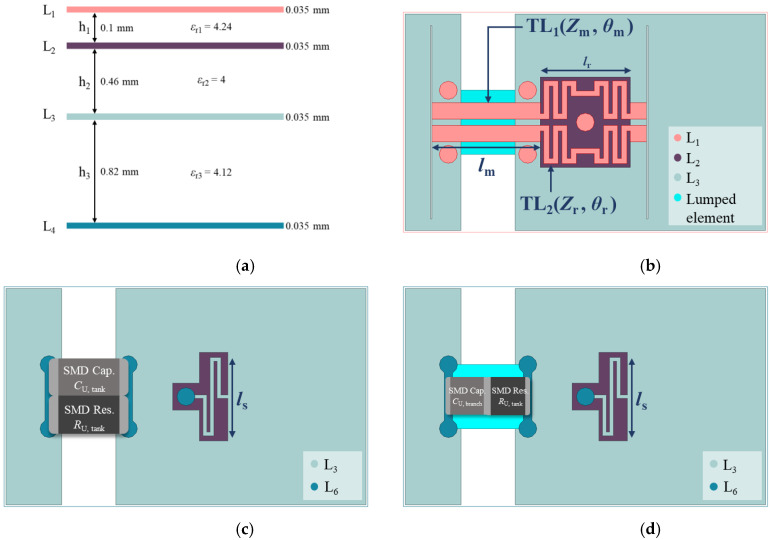
(**a**) The stack of PCB board; (**b**) Top view of the proposed design; (**c**) Bottom view of the proposed design of an RC-tank Unified Circuit; (**d**) Bottom view of the proposed design of an RC-branch Unified Circuit.

In Section 3, we proposed and validated the functional structure of the novel design of A-CMNF, as shown in Figure 6. The stack is a four-layered structure, where L_1_ is used for the signal trace, and L_2_ and L_3_ are used for the return path, as shown in Figure 6a. Each stack height for h1, h2, and h3 is 0.1, 0.46, and 0.82 mm, respectively. The relative permittivity of each stack (*ε*_r1_–*ε*_r3_) is 4.24, 4, and 4.12, and the thickness of all metal layers is 0.035 mm.

The upper three metal layers are used to design an A-CMNF structure, and the bottom layer mounts 0805 SMD components that are 2 × 1.2 mm in size, as shown in Figure 6b,c. The electrical length, *θ*_m_, is about λ/24 (equal to 15° or 0.2618 radians) at 2.45 GHz. The odd-mode characteristic impedance, *Z*_m(odd)_, is about 50 Ω (meanwhile, the even-mode characteristic impedance, *Z*_m(even)_, is about 103.5 Ω), while the line width and spacing are 0.46 and 0.18 mm, respectively. The electrical length, *θ*_r_, is λ/8 (equal to 45° or 0.7854 radians), and its line width and spacing are 0.15 and 0.18 mm, respectively. The odd-mode characteristic impedance, *Z*_r(odd)_, is also about 50 Ω. The relation between structure and characteristic impedance is sorted in Table 1. The commercial design software Advanced design system practically calculates the even-odd-mode characteristic impedance. The other physical and electrical parameters are listed in Table 2. Only *θ*_m_ and *θ*_r_ gave electrical length because those physical dimensions will vary in different PCB stacks. Still, their electrical length is fixed.

**Figure 7:**
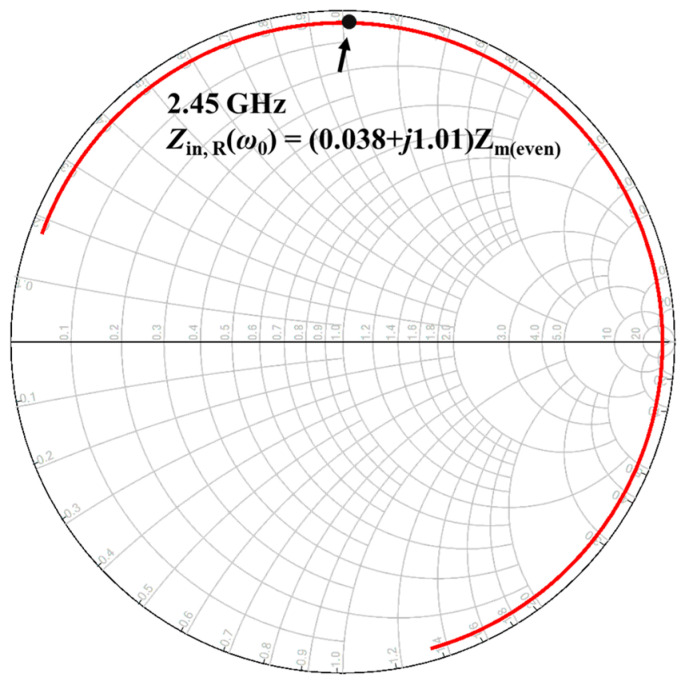
Using Smith Chart validates the input impedance of R-CMNF and checks that it works as an inductive load.

The input impedance of R-CMNF, represented by the Smith Chart, is shown in Figure 7. This validated that it works as an inductive load at the resonance frequency. Note that *Z*_in,R_(*ω*_0_) means the input impedance of the whole structure, and the components *R*_U, tank_, and *C*_U,tank_ can be directly calculated by using (5)–(7). The value of the components is listed in Table 3. We also especially listed the component’s value of the *RC*-branch for simulation validation.

**Figure 8:**
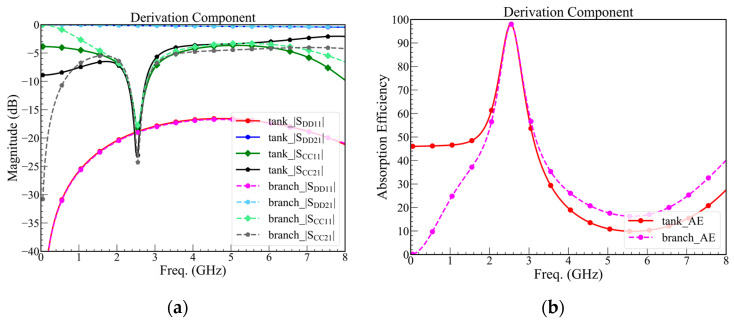
(**a**) Mixed-mode *S*-parameters of the proposed design with derivation components, and (**b**) absorption efficiency of the proposed design with derivation components.

The mixed-mode *S*-parameters are shown in Figure 8a, including *RC*-tank and *RC*-branch, which make sure both are functional. However, we chose the smaller structure because this article aims to propose a compact A-CMNF. Hence, the *RC*-branch is only validated by a simulated and manufactured *RC*-tank.

Both structures are simulated by using the component value listed in Table 3. For both structures, the |*S*_DD21_| is larger than −3 dB until 8 GHz, |*S*_CC21_| and |*S*_CC11_| are smaller than −20 dB at the resonance frequency, and the bandwidth of absorption efficiency over 95% are larger than 80 MHz, which means that the bandwidth is enough to cover the bandwidth of 2.4 GHz Wi-Fi. In addition, (8) is the definition of the absorption efficiency, which is over 95% when the |*S*_CC21_| and |*S*_CC11_| are smaller than −20 dB, as shown in Figure 8b.

**Figure 9:**
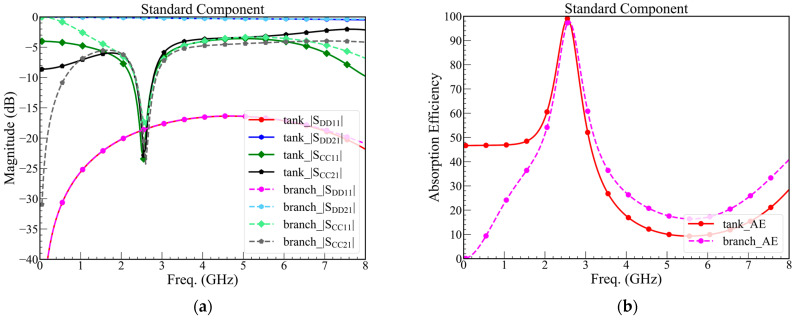
(**a**) Mixed-mode *S*-parameters of the proposed design with standard components, and (**b**) absorption efficiency of the proposed design with standard components.

However, in practice, using the derivation component will increase the cost because that is customized. Thus, alternating the standard component value of the capacitor and resistor will become 51 Ω, 110 Ω, 1.2 pF, and 0.5 pF of *R*_U,branch_, *R*_U,tank_, *C*_U,branch_, and *C*_U,tank_, respectively. Additionally, we use the standard components to simulate and predict the performance of the practical circuit, as shown in Figure 9.

**Figure 10:**
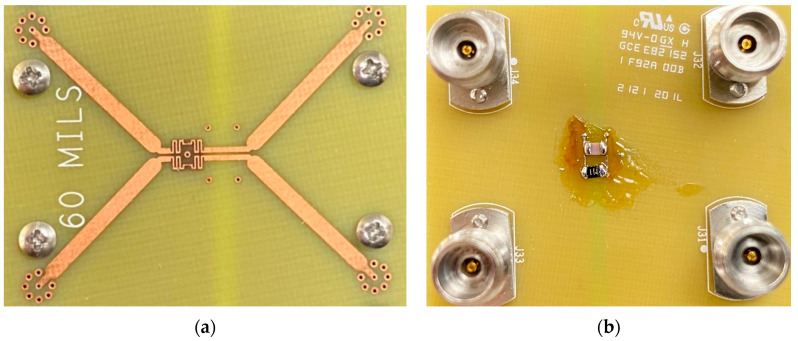
(**a**) Top and (**b**) bottom view of the practical circuit.

Inventec manufactures the practical circuit with four extra single-end transmission lines for the feeding, but it will be de-embed when measuring. The center part of Figure 10a is A-CMNF. Figure 10b is the bottom side, and the standard SMD components are the resistor and capacitor, soldered between two copper lines, in the RC-tank Unified Circuit.

**Figure 11:**
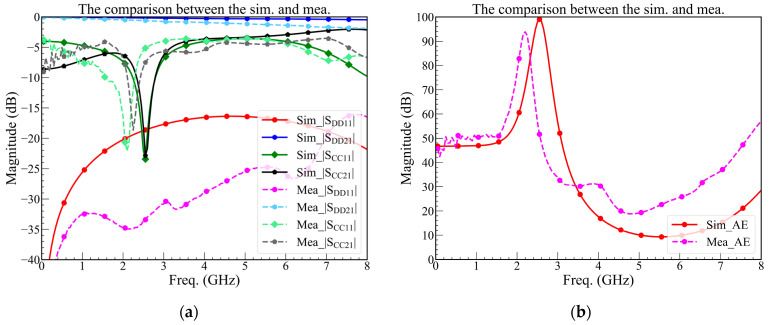
(**a**) The comparison of mixed-mode *S*-parameter of simulation and measurement; (**b**) the comparison of absorption efficiency of simulation and measurement.

The measurement results are compared with the simulation of the *RC*-tank, as shown in Figure 11. The |*S*_DD21_| is met with the simulation and |*S*_DD11_| is smaller than the prediction. The |*S*_CC21_| and |*S*_CC11_| are shifting, but the bandwidth of the absorption efficiency of more than 90% still exceeds 80 MHz. The shift might be an error in the PCB process and welding procedure. The result means the design is functional, but the tolerance of the PCB process and SMD components value must be considered because these will shift the absorption band.

Table 4 shows the comparison of the proposed design with the reference work. The area of the proposed design is the smallest, and the number of the stack layer is the same as in previous works. The common-mode noise absorption bandwidth (CMNA-BW) is about 200 MHz.

## 4. Conclusions

This paper proposed a very compact A-CMNF design than before and gave a precise analysis of the equivalent circuit. Using the precise equivalent circuit analysis to design an A-CMNF in different PCB processes becomes very easy. The other feature of the proposed way is that even if the R-CMNF is a different design, it is still functional, which means that one can freely choose R-CMNF. The features mentioned above are combined in the series Unified Circuit that greatly reduces the size of A-CMNF. Finally, the validation results show that the common-mode noise absorption bandwidth is 200 MHz. The center frequency is shifted because of the PCB process and welding, but it is enough to cover the bandwidth of Wi-Fi working at 2.4 GHz. The proposed work is smaller than the prior-art A-CMNF.

## Figures and Tables

**Table 1:** Characteristic Impedance of Practical Structure.

Line Width/Spacing	Reference Plane ^1^	*Z*_odd_	*Z*_even_
0.15/0.18 mm	L_2_	49.72 Ω	60 Ω
0.46/0.18 mm	L_3_	49.94 Ω	103.5 Ω

^1^ The Matching Stage refers to metal layer three (L3), and the Reflective Stage refers to metal layer two (L2). The different reference plane affects the even-odd-mode characteristic impedance.

**Table 2:** Electrical and Physical Parameters.

*θ*_m_	*θ*_r_	*l*_s_	*l*_m_	*l*_r_
λ/24 (15°)	λ/8 (45°)	2.2 mm	2.54 mm	2.54 mm

**Table 3:** Derivation component value of the unified circuit.

*Z*_U_(*ω*_0_)	*R*_U,tank_	*R*_U,branch_	*C*_U,tank_	*C*_U,branch_
100-*j*105Ω	105 Ω	50 Ω	0.65 pF	1.24 pF

**Table 4:** Area Comparison with Reference of Unidirectional A-CMNF.

Work	Area (*λ*_g_^2^) ^1^	Layer	CMNA-BW ^2^
[9]	0.0042	4	2.0–2.3 GHz
[10]	0.0127	4	2.4–2.5 GHz
[13]	0.0848	2	1.8–3.0 GHz
[15]	0.0358	2	2.33–2.67 GHz
Proposed	0.00316	4	2.1–2.3 GHz

^1^ *λ*_g_ is the guided wavelength at the greatest lower bound of the common-mode noise absorption band; ^2^ both |*S*_CC21_| and |*S*_CC11_| are less than −10 dB.

## Data Availability

Not applicable.
